# Mapping the hippocampal spatial proteomic signature in male and female mice of an early Alzheimer’s disease model

**DOI:** 10.1186/s13293-025-00697-5

**Published:** 2025-05-25

**Authors:** Ana Contreras, Raquel Jiménez-Herrera, Souhail Djebari, Juan D. Navarro-López, Lydia Jiménez-Díaz

**Affiliations:** Neurophysiology & Behavior Lab, Biomedicine Institute (IB-UCLM), School of Medicine of Ciudad Real, University of Castilla-La Mancha, Ciudad Real, 13071 Spain

**Keywords:** MALDI imaging, Alzheimer’s disease, Spatial proteomic, Sex dimorphism, Dorsal/ventral hippocampus, Synaptic plasticity, GSK-3*β*

## Abstract

**Background:**

Hippocampal dysfunction induced by soluble amyloid-*β* oligomers (oA*β*) is an early neuropathological hallmark of Alzheimer’s disease (AD). oA*β* shifts hippocampal synaptic-plasticity induction threshold facilitating long-term depression (LTD) instead of long-term potentiation (LTP, the functional basis of memory), thereby leading to memory deficits in early AD-like amyloidosis mouse models. In this regard, the spatial distribution of the underlying synaptic-plasticity/memory proteome changes in the hippocampus, and potential sex differences, remain unknown. Here we postulated that some protein changes related to synaptic-plasticity and memory may be unique to sex and/or specific to the dorsal or ventral hippocampus −as both regions have distinct functionality and connectivity−, potentially providing sex- and spatial-specific proteomic phenotypes for early AD-amyloidosis interventions.

**Methods:**

An innovative spatial-resolution proteomics study was performed to map whole hippocampal proteome distribution using matrix-assisted laser desorption/ionization (MALDI) imaging mass spectrometry. For this purpose, sixteen adult male and female mouse brains intracerebroventricularly injected with oA*β*_1−42_/vehicle were analyzed. MALDI-imaging RapifleXTM-MALDI-TissuetyperTM TOF/TOF mass spectrometer was used, followed by traditional tandem mass spectrometry (MS/MS) for precise protein identification on tissue.

**Results:**

34 proteins showed significant differences in expression levels due to treatment, sex, or hippocampal location among 234 peptides initially detected; and displayed significant protein-protein interaction (PPI), indicating main functional relationship to LTP/LTD pathways and memory. Thus, 14 proteins related to synaptic-plasticity and/or AD were further studied, showing that most modulated glycogen synthase kinase-3*β* (GSK-3*β*), a protein widely involved in synaptic-plasticity induction threshold regulation towards LTD. Accordingly, hippocampal GSK-3*β* was found to be overactivated in AD-like amyloidosis mice.

**Conclusions:**

We show for the first-time specific sex-dependent synaptic-plasticity proteome changes in dorsal/ventral hippocampi that modulate GSK-3*β* activity. These findings provide new insight into the early amyloidosis pathogenesis in AD and offer valuable, unique proteomic phenotypes as potential biomarkers and targets for early diagnosis and therapy in both sexes.

**Supplementary Information:**

The online version contains supplementary material available at 10.1186/s13293-025-00697-5.

## Abstract (plain language summary)

Previous proteomic studies have shown hippocampal synaptic-plasticity proteins alterations in Alzheimer’s disease. However, the spatial distribution and sex-dependence of these proteomic changes remain unknown. We used MALDI imaging to map the spatial proteomic signatures of the dorsal and ventral hippocampi induced by early amyloidosis in male and female mice. Spatial- and sex- dependent proteome alterations were found functionally related to long-term synaptic plasticity and memory and were associated with GSK-3*β* overactivation, thus providing unique potential biomarkers and targets for early diagnosis and treatment.

**Graphical Abstract:**

**Supplementary Information:**

The online version contains supplementary material available at 10.1186/s13293-025-00697-5.

## Highlights

- oA*β*_1-42_ single *icv.*-injection altered hippocampal proteome in a sex-dependent manner.
- Dorsal and ventral hippocampi showed differences in proteins altered by oA*β*_1-42_.
- PPI showed an involvement of altered proteins in long-term synaptic plasticity and memory.
- Altered proteins modulated GSK-3*β*, causing its overactivation.
- We show a proteomic substrate for early intervention in AD-like amyloidosis.

**Supplementary Information:**

The online version contains supplementary material available at 10.1186/s13293-025-00697-5.

## Graphical abstract

**Figure:**
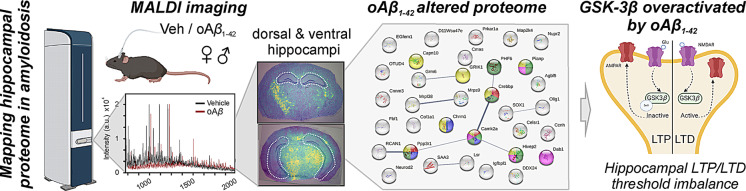

## Background

Alzheimer’s disease (AD) is the most prevalent cause of dementia, accounting for an estimated 60–80% of the 55 million dementia cases worldwide [1]. One of its early hallmarks is the presence of amyloid-*β* (A*β*) peptides [2]. The hippocampus, a critical brain area involved in learning and memory, is among the first regions affected by early amyloidosis, leading to impairments in excitatory/inhibitory (E/I) neurotransmission balance, synaptic plasticity and oscillatory activity, which ultimately result in learning and memory disfunction [3–5].

Amyloidogenic processing of amyloid precursor protein (APP) causes the aggregation of different A*β* species, being the 42 amino acid-long amyloid-*β* (A*β*_1−42_) the dominant form in amyloid plaques in AD patients [6, 7]. However, AD pathophysiology starts decades before the formation of amyloid plaques, during the stage when A*β* remains soluble rather than accumulated [8]. A*β*_1−42_ monomers are highly prone to aggregation, forming a wide range of soluble oligomers, from dimers to trimers, which eventually fibrillate into A*β* plaques [9, 10]. Soluble A*β*_1−42_ oligomers (oA*β*_1−42_) are considered the major toxic agents in early AD, leading to the initial loss of excitatory synapses and inhibition of synaptic plasticity both in vivo and in vitro [11–13].

To study these early stages of AD, our group and others have previously validated a murine model of intracerebroventricular (*icv.*) oA*β*_1−42_ administration, showing E/I imbalance, deficits in long-term synaptic potentiation (LTP) induction, disruption of neural oscillatory synchronization and impairments in learning and memory, in both male and female mice [14–18].

Several omics techniques have been widely used to obtain an unbiased insight into the molecular changes associated with AD progression, such as genomics [19], proteomics [20, 21] and metabolomics [22]. In fact, a specific plasma proteomic profiling has shown stage-dependent dysregulations in AD [23], highlighting the importance of biomarker discovery for early identification of AD risk factors based on proteomic profile. However, most of these techniques are carried out in cerebrospinal fluid [24], plasma [23] or brain homogenates [25–28], which lack spatial information on the distribution of those potential biomarkers in the brain. Spatial information is key, as the distribution of Aβ deposits and tau tangles in the brain correlates with the regional atrophy and cognitive decline at different AD stages [29–32]. In this context, matrix-assisted laser desorption/ionization (MALDI) imaging mass spectrometry stands out in the last decade as a particularly powerful tool that allows molecular mass determination of analytes directly on the tissue, with spatial resolution. Using MALDI imaging, previous works have revealed differential distributions of lipids, glycomes and metabolites in different brain areas in both AD patients and murine models [33–36]. A recent study has shown alterations in proteins related to synaptic function and neurodegeneration, specifically in the cortex, the ventricular zone and the corpus callosum of neonatal 5xFAD mice using MALDI imaging [37]. However, the differential distribution of proteins in the hippocampus during early amyloidosis remains unexplored, despite its functional, molecular and connectivity heterogeneity [38–40].

Here, for the first time, the hippocampal proteome of healthy and early AD-like amyloidosis male and female mice was investigated with spatial resolution, and potential proteomic expression differences between dorsal and ventral hippocampal areas were analyzed, providing cutting-edge data on the specialization of the hippocampal proteome and identifying valuable potential biomarkers for early diagnosis and treatment.

## Methods

### Animals

Twenty-three female and twenty-five male C57BL/6 adult mice (12–24 weeks old; 20–30 g) were used (RRID: MGI:5,656,552; Charles River, USA). The mice were housed under 12-hour (h) light/dark cycles with unrestricted access to food and water and controlled temperature (21 ± 1ºC) and humidity (50 ± 7%). Mice were housed in same-sex groups of 3–5 per cage before surgery, and individually afterwards. Environmental enrichment elements were provided. All experimental procedures were carried out at the same time interval in both female and male mice to minimize circadian rhythm interferences.

All experimental procedures were reviewed and approved by the Ethical Committee for Use of Laboratory Animals of the University of Castilla-La Mancha (PR-2021-12-21) and conducted according to the European Union guidelines (2010/63/EU) and the Spanish regulations for the use of laboratory animals in chronic experiments (RD 53/2013 on the care of experimental animals: BOE 08/02/2013).

### Surgery for generation of the early hippocampal AD-like amyloidosis mouse model

Anesthesia was induced with 4% isoflurane (#13400264, ISOFLO, Proyma S.L., Spain) administered via a calibrated R580S vaporizer (RWD Life Science; flow rate: 0.5 L/min O_2_). During the procedure, 1.5% isoflurane was delivered constantly for anesthesia maintenance, after which intramuscular buprenorphine (0.01 mg/kg; #062009, BUPRENODALE, Albet, Spain) and a healing cream (Blastoestimulina; Almirall, Spain) were administered to accelerate recovery and decrease animal suffering.

Mice were implanted with a blunted, stainless steel, 26-G guide cannula (Plastics One, USA) in the left ventricle (1 mm lateral and 0.5 mm posterior to bregma; depth from brain surface, 1.8 mm) [41] for *icv*. administration of oA*β*_1−42_. The final position of the cannula was determined by Nissl staining after brain tissue collection [18].

This model has been extensively characterized and validated by our group and others to study early AD-like neuropathology at molecular, synaptic, network and behavioral levels [14–18]. As previously described [18], monomeric A*β*_1−42_ (#AB120301; Abcam, UK) was dissolved in phosphate-buffered saline (PBS) and incubated 4–6 h at 37ºC to form soluble oA*β*_1−42_ before administration [42]. The formation of oA*β*_1−42_ was previously demonstrated by western blot by incubating with a specific antibody and quantifying the molecular weight band corresponding to the oligomers [18]. Furthermore, it has been shown that after *icv*. injection, oA*β*_1–42_ diffuses into both hippocampi, initially reaching the dorsal region and ultimately spreading throughout the entire hippocampus, generating an acute hippocampal amyloidosis, whose functional effects last more than two weeks [14–18].

**Fig. 1:**
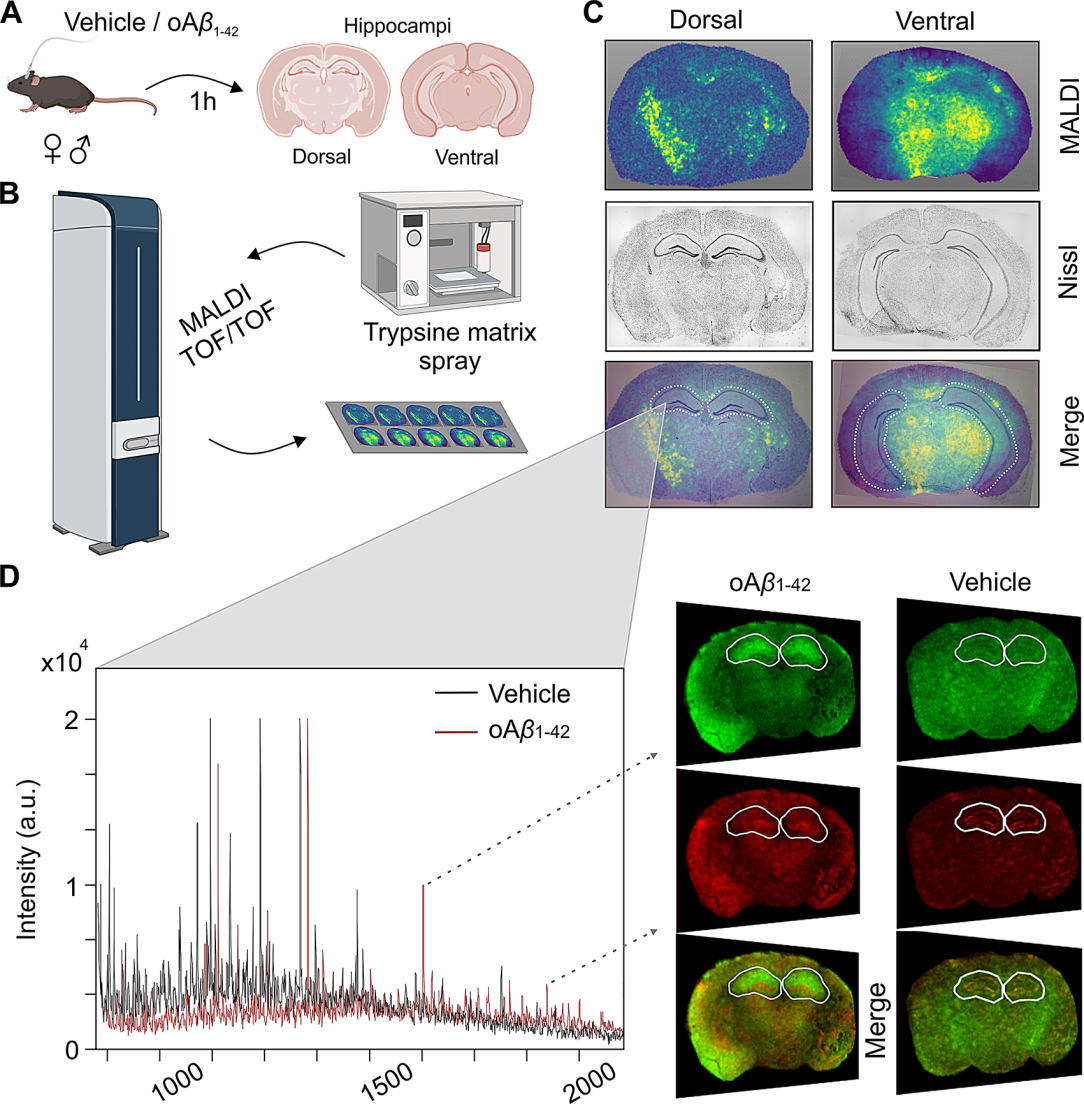
Experimental design. **(A)** Schematic illustration of *icv*. injection of either vehicle or oA*β*_1−42_ in male and female mice and dissection of dorsal- and ventral-containing hippocampal Sect. 1 h later. **(B)** Schematic representation of trypsin and CHCA matrix spray application with a HTX TM SprayerTM and subsequent MALDI imaging acquisition using a RapifleXTM MALDI TissuetyperTM TOF/TOF mass spectrometer. **(C)** MALDI images showing protein markers (top), Nissl staining of consecutive tissue sections (middle, in gray) and overlay of the two (bottom), with delimitation of dorsal (left) and ventral (right) hippocampi in white (dashed line). **(D)** Representative peptide spectrum (left) of hippocampal tissue measured by MALDI imaging from vehicle (black) and oA*β*_1−42_treated (red) mice with examples (right) of two selected peptides shown in tissue sections (green and red) and its merge

After a 1–2 weeks recovery period, freely moving animals received a 3 µL *icv.* injection of either 1 µg/µL of oA*β*_1−42_ or vehicle (PBS) through a 33-G internal cannula within the implanted guide cannula and protruding 0.5 mm into the ventricle, using a motorized Hamilton syringe at a rate of 0.5 µL/min (Fig. 1A). After administration, the internal cannula was not removed for an extra minute to avoid backflow. The dose was selected based on previous studies that demonstrated its efficacy and safety [14–16, 18].

### Post-injection brain tissue collection and preparation for MALDI MS imaging

1 h after treatment, animals were deeply anesthetized with halothane (Fluothane; AstraZeneca, UK) and decapitated as described previously [18], and fresh brains were extracted and dissected (Fig. 1A). This time point was chosen based on our previous experiments extensively showing evident spatial and habituation memory impairments without sex dimorphism (by Barnes maze, object location memory and open field habituation testing) in our mouse model of early acute hippocampal AD-amyloidosis 1 h post-*icv*. injection of oA*β*_1−42_ [18]. Synaptic plasticity impairments (LTP disruption and LTD induction) were also evident at this time point [14–18].

After dissection, the medial part of the brain, containing both hippocampi, was fresh frozen at -80ºC until further use. Hippocampal-containing sections were cut into 10 μm thick coronal slices using a CM3050 S cryostat (Leica, Germany) at -20ºC, and thaw-mounted onto indium tin oxide (ITO) coated glass slides (Bruker Daltonics, Germany) [43]. Each treatment group included four female and four male mice, with four slices from each animal used for MALDI imaging experiments, half of them containing the dorsal hippocampus (Bregma − 1.22 to -1.94 mm) and the other half the ventral hippocampus (Bregma − 2.54 to -3.28 mm) [41] (Fig. 1A).

To remove most of the lipids from the sections, slices were washed in Carnoy’s solution (6 ethanol: 3 chloroform: 1 acetic acid; all from Thermo Fisher, USA) [44], and allowed to dry. Then, a solution containing 25 µg/mL of sequencing grade trypsin (Promega, USA) diluted in 20 mM ammonium bicarbonate (Thermo Fisher, USA) was uniformly applied onto the tissue sections using a HTX TMSprayerTM (HTX Technologies, USA) in 15 layers under the following conditions: nozzle height 40 mm, nitrogen pressure 10 psi, spray flow rate of 10 µL/min, temperature 30ºC, z-arm velocity 750 mm/min, track spacing 2 mm, crisscross pattern and drying time 0 s. Samples were then incubated overnight with trypsin in a humidity chamber at 37ºC.

After air-drying, the slides were coated with α-Cyano-4-hydroxycinnamic acid (CHCA, 7 mg/mL; Bruker Daltonics, Germany) in 70% acetonitrile (ACN)/1% trifluoroacetic acid (TFA) using the same sprayer (HTX Technologies, USA). The sprayer settings were: 8 passes, nozzle height 40 mm, nitrogen pressure 10 psi, flow rate of 0.1 mL/min, temperature 75ºC, z-arm velocity 1200 mm/min, track spacing 3 mm, crisscross pattern and drying time 0 s. Following air-drying, the samples were immediately analyzed using MALDI imaging.

### MALDI imaging acquisition and analysis

All imaging analysis were performed using a RapifleXTM MALDI TissuetyperTM TOF/TOF mass spectrometer (Bruker Daltonics, Germany) equipped with a Smartbeam™ 3D laser (Fig. 1B).

Mass measurements were performed in reflector positive ion mode in the m/z range of 600 to 3500 Da, with a spatial resolution of 50 μm. External calibration was performed using Peptide Calibration Standard Kit II (Bruker Daltonics, Germany). For histological annotation and precise hippocampal delineation, standard Nissl staining was applied to consecutive tissue sections and photographed using an Axio Imager.M2 microscope (Zeiss, Germany) (Fig. 1C).

Visualization and statistical analysis of the peak list were performed with FlexImaging and SCiLS Lab software (version 2024b Pro; Bruker Daltonics, Germany). Data from the tissue sections were imported to SCiLS Lab. The processing steps included baseline subtraction (Top-hat filter), normalisation (Total Ion Current algorithm), and spatial denoising (weak). Peaks were aligned to the mean spectrum by centroid matching. Average spectra, representative of the whole measurement regions and ROIs, were generated to display differences in the peptide profiles (Fig. 1D). Intensity values for each m/z peak were exported to Excel for further calculations.

### MS/MS of digested tissue sections

Once each m/z was analysed, the precise identification of the corresponding protein of the ones showing significant differences between the groups was performed by spatially targeting and sequencing peptides using traditional tandem mass spectrometry (MS/MS) approaches directly on the tissue. The resulting MS/MS spectra were submitted to a MASCOT (Matrix Science, USA) database search engine using BioTools software (Bruker Daltonics, Germany) to match tryptic peptide sequences to their respective intact proteins. The search was performed with a parent ion tolerance of 100 ppm and a fragment ion tolerance of ± 0.3 Da against the *Mus musculus* database. The search criteria also included up to two missed cleavages and variable modifications, including protein N terminus acetylation, histidine/tryptophan oxidation, and methionine oxidation.

### Study of protein-protein interaction (PPI)

To generate a functional association network of the identified proteins and calculate the protein-protein interaction (PPI) enrichment *p*-value, STRING v11.5 database (https://string-db.org/) was used. A significant PPI value means that the proteins included in the study have more interactions among themselves than would be expected for a random set of proteins of similar size, drawn from the genome, and indicates that the proteins are partially biologically connected as a group [45]. Statistics were performed using the STRING database statistics package, with a default confidence level cutoff of 0.4.

### Western blot

To prove the reproducibility of our proteomic results, some of the proteins identified by MALDI imaging analysis as significantly altered, as well as a results-related protein of interest, were additionally measured by Western blot. For this validation, one up- and one down-regulated protein were selected based on the availability of commercial validated antibodies.

Hippocampal tissue samples (*n* = 5–9 per group) were homogenized in ice-cold RIPA lysis buffer (50 mM Tris-HCl pH 7.4; 150 mM NaCl; 0.1% Tx100; 0.5% sodium deoxycholate; 0.1% SDS) with protease and phosphatase inhibitors (all from Roche Diagnostics, Germany). Afterwards, protein concentration in each sample was quantified using the Pierce BCA Protein assay kit (Thermo Fisher, USA) and all samples were adjusted to contain 1 ug/ul of protein. Equal amounts of protein (30 µg) were mixed with Laemmli buffer (Bio-rad, USA), loaded on a sodium dodecyl-sulfate polyacrylamide gel electrophoresis (SDS-PAGE) and subjected to electrophoresis. Proteins were transferred to nitrocellulose membranes (Bio-rad, USA) by using a transblot apparatus (Bio-Rad, USA). Membranes were blocked with 5% dried skimmed milk powder in Tween-PBS for 1 h. Primary antibodies against each protein of interest were applied at appropriate dilutions overnight at 4ºC (Table 1). After washing, corresponding secondary antibodies (anti-rabbit or anti-mouse HRPconjugated, #AP307 and #AP130P respectively; Sigma-Aldrich, USA) were added for 1 h at a dilution of 1/5000. Blots were washed, incubated in enhanced chemiluminescence reagent (ECL Prime; Bio-rad, USA), and developed using the G: BOX Chemi XX6 gel documentation system (Syngene, India).

**Table 1:** Characteristics of primary antibodies used to measure protein levels by western blot

Protein	Supplier	Host species	Dilution	Molecular weight
RCAN1	Proteintech	Rabbit	1.2000	25–30 kDa
GluR5	Proteintech	Rabbit	1.2000	80 KDa
pGSK-3*β* Ser9	Proteintech	Mouse	1.2000	48 kDa
GSK-3*β*	Proteintech	Rabbit	1.2000	48 KDa
*β*actin	Sigma-Aldrich	Mouse	1.5000	42 kDa

An antibody against *β*actin (Table 1) was used as a loading control. For blot quantification, density of each band was determined using ImageJ software (ImageJ, USA). Values were expressed as the ratio of protein of interest/*β*actin, or phosphorylated protein/total protein when appropriate, and in percentage of control (vehicle) group (100%).

### Statistical analysis

Data were represented as mean ± SEM. Before analysis, outliers were identified by the ROUT method with a Q cutoff of 1%. Data were analyzed by two- or threeway ANOVA, using treatment and sex or treatment, sex and hippocampal region as factors, respectively, and followed by Tukey’s *post-hoc* analysis. When comparing only two groups, unpaired two-tailed Student t test was used. Statistical significance was set at *p* < 0.05, and a fold change (FC) ≤ 0.66 (downregulated) or ≥ 1.5 (upregulated). All analyses were performed using SPSS software v.24 (RRID: SCR_002865; IBM, USA) and GraphPad Prism software v.8.3.1 (RRID: SCR_002798; Dotmatics, USA). Final figures were prepared using CorelDraw X8 Software (RRID: SCR_014235; Corel Corporation, Canada).

### Experimental design and statistical rationale

For MALDI imaging, sixteen C57BL/6 adult mice were used, with oA*β*_1−42_ administered to half of them and vehicle to the other half. All experiments were carried out in four independent biological replicates from each animal, with two containing the dorsal hippocampus and two containing the ventral hippocampus. The number of animals was selected to ensure sufficient statistical power while adhering to the 3Rs principle. Samples were run in a blinded manner for MALDI imaging and MS/MS. Data points were excluded if statistical analysis identified them as outliers. Statistical significance was set at *p* < 0.05, and a FC ≤ 0.66 or ≥ 1.5. This FC range was chosen as it shows the most strongly changed peptides. Analysis was performed with either two- or threeway ANOVA or Student t test when appropriate based on the number of variables and groups being compared.

## Results

### Differentially expressed proteins across the whole hippocampus: early amyloidosis and sexually dimorphic differences in synaptic plasticity-related proteins

**Fig. 2:**
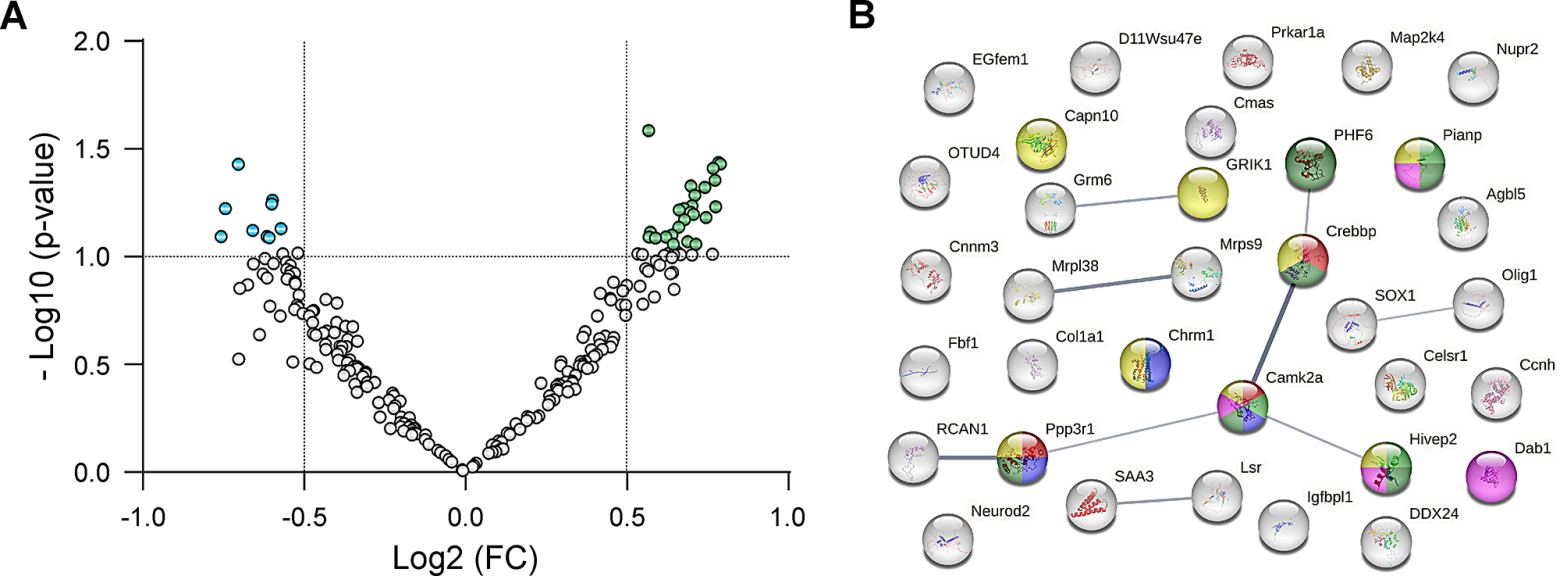
Hippocampal proteins significantly affected by treatment with oA*β*_1−42_, sex or both, and interaction among them. **(A)** Volcano plot showing 9 downregulated (blue points) and 25 upregulated proteins (green points) with a fold change ≥ 1.5. **(B)** Proteinprotein interaction (PPI) of the differentially expressed proteins. A significant PPI (*p* < 0.05), according to STRING database, was found. Each sphere represents a single protein (see details in Table 2). Colored proteins are included in the leading significantly enriched pathway and phenotypes (in order of strength): long term potentiation (red), abnormal response to social novelty (light green), reduced long term depression (blue), abnormal social investigation (dark green), abnormal dentate gyrus morphology (pink) and abnormal synaptic transmission (yellow). Lines represent protein − protein interactions, and line thickness indicates the strength of data supporting the interaction (Table 3)

MALDI imaging quantification detected 234 peptides. Of these 234, 34 showed significant differences (≤ 0.66 or ≥ 1.5-fold change) between groups due to either treatment, sex, hippocampal area, or interactions between them (Fig. 2A). The volcano plot showed that most altered proteins were upregulated (74%) and only a few were downregulated (26%). The specific proteins corresponding to those 34 peptides were identified using MS/MS directly on the tissue (Table 2).

**Table 2:** Differentially expressed proteins due to oA*β*_1−42_ treatment and/or sex in whole, dorsal and ventral hippocampus

m/z (± 0.2Da)	Protein name	Abbreviation	Uniprot KB ID	Score	Coverage (%)	Tandem mass spectrometry sequence data	Significant differences
699.451	Nuclear protein 2	Nupr2 / Nupr1L	**Q497P3**	163	20	PSVSGPR	Sex-effect
700.449	Collagen alpha-1(I) chain	Col1a1	*P11087*	15	5	EGGKGPR	Sex-effect
704.39	EGF-like and EMI domain-containing protein 1	EGfem1	*Q8C088*	230	16	QGCSGPR	Treatment-effect
720.402	cAMP-dependent protein kinase type 1-alpha	Prkar1a	*Q9DBC7*		1	TMAALAK M2: Oxidation	Sex-effect
739.407	Calpain-10	Capn10	*Q9ESK3*	233	17	PSGGEHR	Sex-effect
745.442	ATP-dependent RNA helicase DDX24	DDX24	*Q9ESV0*	140	15	STGMPPR	Treatment-effect
747.438	Uncharacterized protein C17orf80 homolog	D11Wsu47e	*Q6PIX9*	163	25	MGAAEPR 1: Oxidation (M)	Treatment-effect
776.419	PHD finger protein 6	PHF6	*Q9D4J7*	228	19	SSPNDTR	Treatment- and sex-effects
816.474	OTU domain-containing protein 4	OTUD4	*B2RRE7*	517	22	SGMGDGHR	Sex-effect
828.446	Transcription factor SOX-1	SOX1	*P53783*	325	30	APCPGDLR	Sex-effect
873.539	Cadherin EGF LAG seven-pass G-type receptor 1	Celsr1	*O35161*	578	21	LSVSSGPAR	Sex-effect
928.509	Dual specificity mitogen-activated protein kinase 4	Map2k4	*P47809*		18	PSGQIMAVK	Sex-effect
947.514	Regulator of calcineurin 1	RCAN1	*Q9JHG6*	282	41	MEDGVAGPR 1: Oxidation (M)	Treatment- and sex-effects
953.549	Oligodendrocyte transcription factor 1	Olig1	*Q9JKN5*	776	23	AEAPLAEPR	Sex-effect
954.547	CREB-binding protein	Crebbp / Cbp	*P45481*	701	31	MPNVQPPR 1: Oxidation (M)	Treatment- and sex-effects
985.574	Serum amyloid A3	SAA3	*P04918*		25	RGPGGAWAAK W7: Oxidation	Sex-effect
1001.586	Disabled homolog 1	Dab1	*P97318*	1151	23	SSPQSDKPR	Sex-effect in ventral hippocampus
1006.524	Metal transporter CNNM3	Cnnm3 / Acdp3	*Q32NY4*	777	26	LRAEAGHPR	Sex-effect
1066.582	Muscarinic acetylcholine receptor M1	Chrm1 / M1 mAChR	*P12657*	1089	19	CCRCCRAPR	Sex*treatment interaction in ventral hippocampus
1067.579	28 S ribosomal protein S9, mitochondrial	Mrps9	*Q9D7N3*	1662	21	QAGLLTPDPR	Sex-effect
1143.649	Lipolysis-stimulated lipoprotein receptor	Lsr / Ildr3 / Lisch7	*Q99KG5*	724	35	SSPPSSGRRGR	Sex-effect in dorsal hippocampus
1144.647	Cytosolic carboxypeptidase-like protein 5	Agbl5 / Ccp5	*Q09M02*	193	33	SSPPTRRGMR	Sex-effect in ventral hippocampus
1192.583	Human immunodeficiency virus type I enhancerbinding protein 2	Hivep2	*Q3UHF7*	579	51	EILPGSRAPPR	Sex-effect
1193.681	PILR alpha-associated neural protein	Pianp / Panp	*Q6P1B3*	4377	30	PPCVRGGPSAPR	Sex-effect in ventral hippocampus
1386.723	Glutamate receptor ionotropic, kainate 1	GRIK1 / GluR5 / GluK1	*Q60934*	199	28	WSMERLQAPPR 3: Oxidation (M)	Treatment-effect
1430.819	Fas-binding factor 1	Fbf1	*A2A870*	380	44	PTVASSEGRQSRR	Sex-effect in ventral hippocampus
1558.16	Metabotropic glutamate receptor 6	GRM6 mGluR6	*Q5NCH9*	1091	41	CPGGVPPLRAAPPER N-Term: Acetyl	Treatment-effect
1643.864	Insulin-like growth factor-binding protein-like 1	Igfbpl1	*Q80W15*	1347	53	DGPCEFAPVVLMPPR 12: Oxidation (M)	Treatment-effect
1736.649	Calcium/calmodulin-dependent protein kinase kinase 2	CaMKK2	*Q8C078*	313	88	GGPCVESWGAPAPGSPPR W8: Oxidation	Sex-effect in dorsal and ventral hippocampus
1773.964	Cyclin-H	Ccnh	*Q61458*	234	47	PAMPRSVVGTACMYFK 13: Oxidation (M) PAMPRSVVGTACMYFK 3: Oxidation (M)	Sex in ventral hippocampus
1790.966	39 S ribosomal protein L38, mitochondrial	Mrpl38	*Q8K2M0*	1644	34	ETDPKDKIDIGLPPPR	Treatment-effect in ventral hippocampus
1794.0	Neurogenic differentiation factor 2	Neurod2 / Ndrf	*Q62414*	200	41	GDAPPQPPPAPGSGAPGPAR	Treatment-effect
2170.068	N-acylneuraminate cytidylyltransferase	Cmas	*Q99KK2*	1083	68	MDALEKGAVTSGPAPRGRPSR 1: Oxidation (M)	Treatment-effect
2574.7	Calcineurin subunit B type 1	Ppp3r1/Cnb	*Q63810*		9	MGNEASYPLEMCSHFDADEIKR M1, M11: Oxidation	Sex-effect in ventral hippocampus

**Table 3:** Specific confident score of each PPI, visually represented as line thickness, retrieved form STRING database

Node 1	Node 2	Score
Crebbp	Camk2a	0.915
RCAN1	Ppp3r1	0.810
Mrps9	Mrpl38	0.740
SAA3	Lsr	0.540
Grm6	GRIK1	0.519
PHF6	Crebbp	0.463
Hivep2	Camk2a	0.453
Ppp3r1	Camk2a	0.440
SOX1	Olig1	0.416

When comparing treated and vehicle groups, differences were found in 13 proteins, while the remaining 21 proteins showed differences when comparing males and females (Table 2).

According to the STRING database analysis, there was a significant interaction (PPI *p*value = 0.04) among the differentially expressed proteins, being the leading significantly enriched pathway and phenotypes, in order of strength: LTP, abnormal response to social novelty, reduced long term depression (LTD), abnormal social investigation, abnormal dentate gyrus morphology and abnormal synaptic transmission (Fig. 2B). Based on an extensive review of existing literature, 14 proteins related to synaptic plasticity and/or AD were selected to further study: RCAN1, Crebbp, GluR5, mGluR6, Igfbpl1, Neurod2, SAA3, Hivep2, Lsr, CaMKK2, M1 mAChR, Dab1, Pianp and Ppp3r1.

**Fig. 3:**
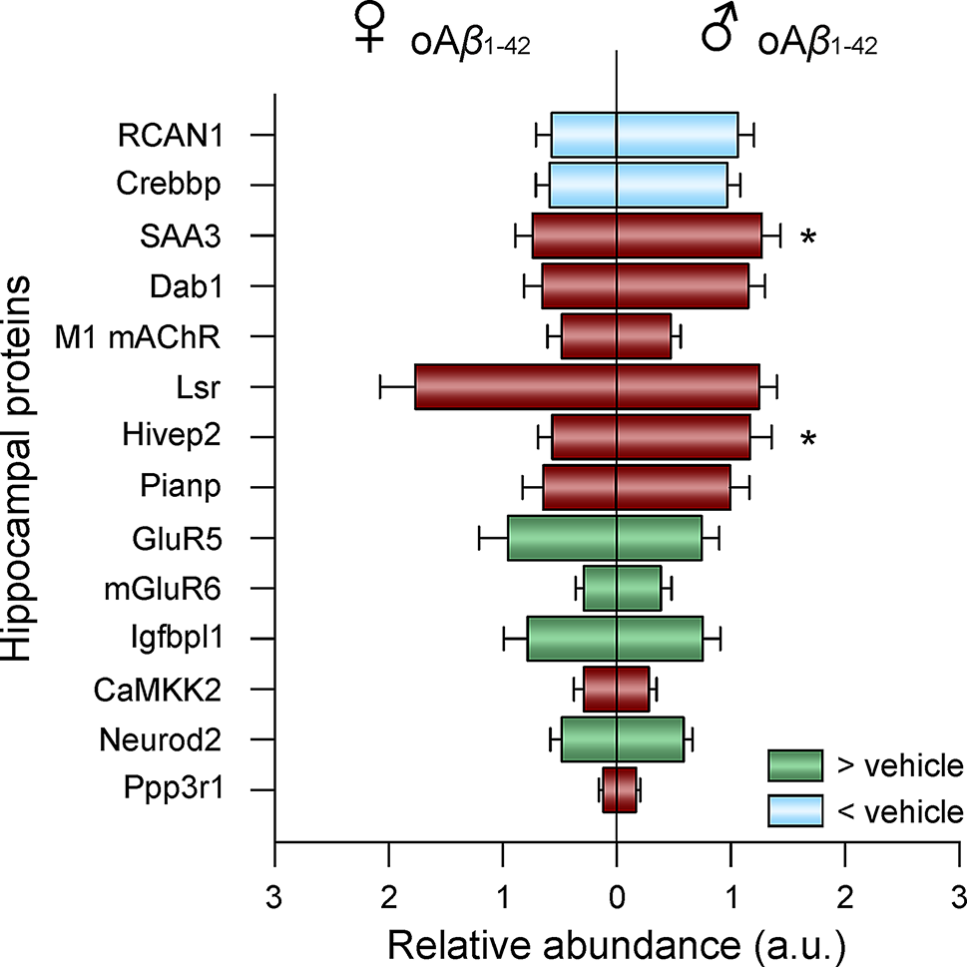
Memory related proteins altered in the whole hippocampus of oA*β*_1−42_ treated female (left) and male (right) mice. Blue indicates downregulation, while green indicates upregulation compared to vehicle of the corresponding sex. Red indicates no difference from the control. Data are expressed as mean ± SEM. * *p* < 0.05 male vs. female within the oA*β*_1−42_ treated animals. A.u., arbitrary units; oA*β*_1−42_, Amyloid*β*_*1−42*_ oligomers; ♀, female; ♂, male

To map the distribution of the selected 14 proteins across the whole hippocampus, MALDI imaging protein expression data for each protein were analysed (Fig. 3). First, the effect of acute hippocampal amyloidosis was studied by analyzing changes induced by oA*β*_1−42_ administration. Data showed a downregulation of RCAN1 (treatment effect: F_(2,77)_ = 5.306, *p* = 0.0069) and Crebbp (treatment effect: F_(2,77)_ = 3.777, *p* = 0.0272) due to oA*β*_1−42_ injection. Conversely, an upregulation of GluR5 (treatment effect: F_(2,79)_ = 4.102, *p* = 0.0202), mGluR6 (treatment effect: F_(2,73)_ = 7.397, *p* = 0.0012), Igfbpl1 (treatment effect: F_(2,78)_ = 6.314, *p* = 0.0029) and Neurod2 (treatment effect: F_(2,80)_ = 6.939, *p* = 0.0017) was observed when comparing treated and control animals.

Furthermore, regarding potential protein differences due to sexual dimorphism, when comparing oA*β*_1−42_ treated females vs. males, differences were found in SAA3 (sex effect: F_(1,77)_ = 8.501, *p* = 0.0046) and Hivep2 (sex effect: F_(1,51)_ = 4.379, *p* = 0.0414), being both of them higher in males than females. However, no sex-differences in these proteins were observed in the vehicle group, suggesting that these are not physiological sex differences but rather a differential effect of oA*β*_1−42_ between males and females.

In order to validate the expressional changes of proteins acquired by the MALDI imaging analyses, Western blot analyses were conducted with two selected altered proteins: one up-regulated (GluR5) and one down-regulated (RCAN1; Additional file 1).

### Differentially expressed proteins in the dorsal and ventral hippocampus: early amyloidosis and sexually dimorphic differences in synaptic plasticity-related proteins

To further explore the spatial distribution of the altered proteins taking advantage of this cutting edge technology, data were segmented to separately analyze the dorsal and ventral hippocampus, as many studies have shown molecular and functional differences between these regions [38–40].

**Fig. 4:**
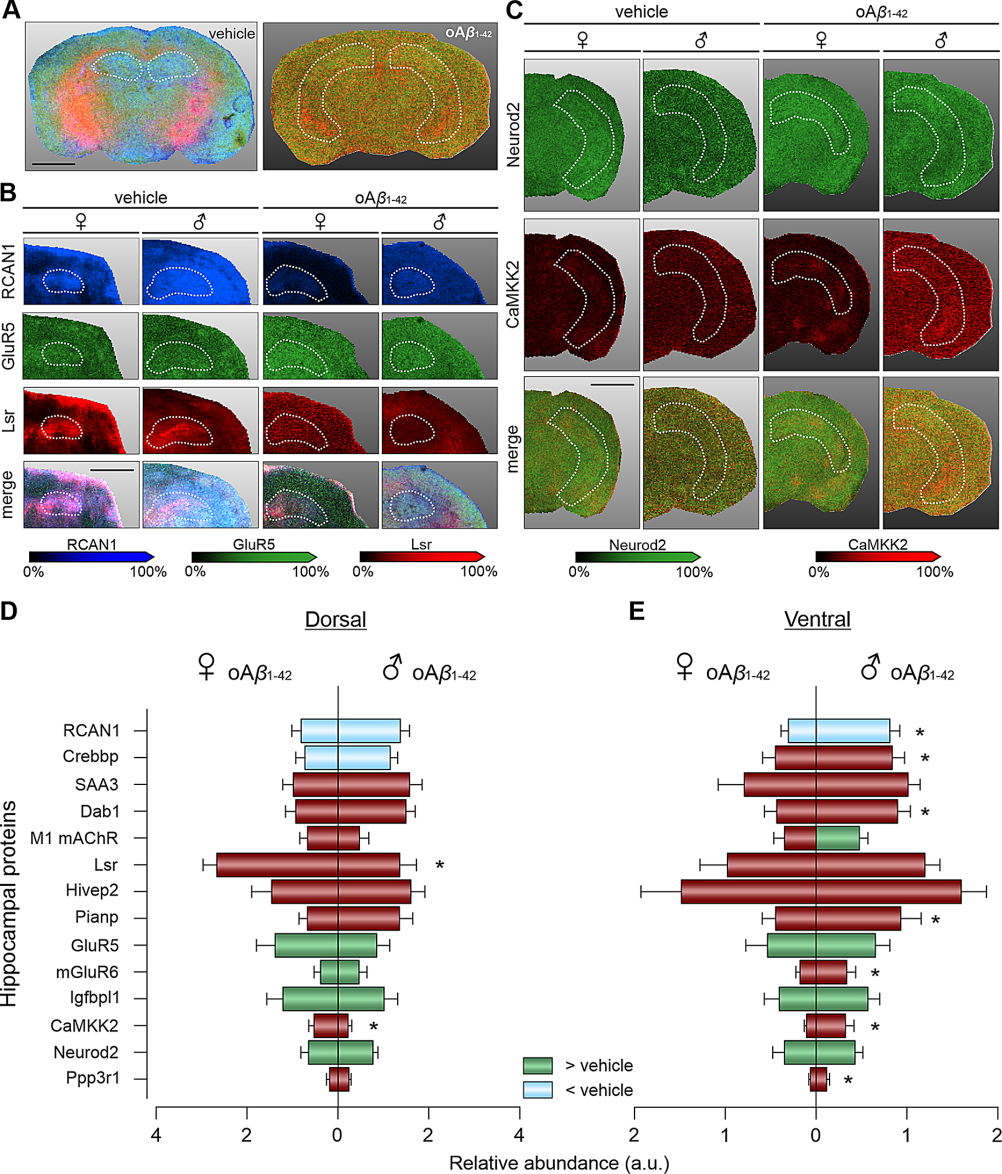
Memory related proteins altered in different hippocampal areas of oA*β*_1−42_-treated female and male mice. **(A)** Examples of MALDI images of dorsal (left) and a ventral (right) tissue sections with delimitation of the hippocampi in white (dashed line) scale bar = 2 mm. **(B)** Representative MALDI images of the dorsal hippocampus with selected peptides (Blue: RCAN1; Green: GluR5; Red: Lsr; and its merge) in vehicle (left) and oA*β*_1−42_ (right) treated animals of both sexes. Scale bar = 2 mm **(C)** Representative MALDI images of the ventral hippocampus with selected peptides (Green: Neurod2; Red: CaMKK2; and its merge) in vehicle (left) and oA*β*_1−42_ (right) treated animals of both sexes. Scale bar in B (2 mm) also applies for images in C **(D)** Expression of proteins in the dorsal hippocampi of oA*β*_1−42_ treated female (left) and male (right) mice. **(E)** Expression of proteins in the ventral hippocampi of oA*β*_1−42_ treated female (left) and male (right) mice. Expression levels are expressed as relative abundance of the specific protein vs. vehicle. Blue indicates downregulation, while green indicates upregulation compared to vehicle of the corresponding sex. Red indicates no difference from the control vehicle. Data are expressed as mean ± SEM. * *p* < 0.05 male vs. female within the oA*β*_1−42_ treated animals. a.u., arbitrary units; oA*β*_1−42_, Amyloid*β*_*1−42*_ oligomers; ♀, female; ♂, male

In the dorsal hippocampus (Fig. 4A, B, D), two-way ANOVAs revealed treatment effects in the same proteins that showed differences in the whole hippocampus (RCAN1: F_(1,22)_ = 5.281, *p* = 0.0314; Crebbp: F_(1,21)_ = 5.92, *p* = 0.024; GluR5: F_(1,22)_ = 4.211, *p* = 0.05; mGluR6: F_(1,21)_ = 7.348, *p* = 0.0131; Igfbpl1: F_(1,22)_ = 7.535, *p* = 0.0118; Neurod2: F_(1,24)_ = 6.019, *p* = 0.0218), with a downregulation of the first two proteins and an upregulation of the last four proteins in oA*β*_1−42_ mice. Regarding sex effects in this area, differences were found only in Lsr (F_(1,21)_ = 9.575, *p* = 0.0055) and CaMKK2 (F_(1,23)_ = 5.957, *p* = 0.0228) between females and males after oA*β*_1−42_ injection, with no differences observed between female and male vehicles.

Interestingly, there were less differences due to treatment and more sex effects in the ventral hippocampus (Fig. 4A, C, E). Specifically, two-way ANOVA revealed a downregulation of RCAN1 (F_(1,24)_ = 6.465, *p* = 0.0179) as well as an upregulation of GluR5 (F_(1,25)_ = 4.521, *p* = 0.0435), Igfbpl1 (F_(1,26)_ = 5.882, *p* = 0.0225) and Neurod2 (F_(1,27)_ = 5.756, *p* = 0.0236) for oA*β*_1−42_-treated mice compared to controls. One protein, M1 mAChR, showed an interaction effect between sex and treatment (F_(1,27)_ = 5.634, *p* = 0.025). Thus, in vehicle-treated animals, females showed higher M1 mAChR expression than males (t_13_ = 2.650, *p* = 0.02). However, after oA*β*_1−42_ treatment, this sex effect disappeared, and M1 mAChR was upregulated specifically in treated males compared to male controls (t_14_ = 2.395, *p* = 0.031). Additional sex differences in the ventral hippocampus were found in RCAN1 (F_(1,24)_ = 18.07, *p* = 0.0003), Crebbp (F_(1,27)_ = 7.552, *p* = 0.0106), Dab1 (F_(1,27)_ = 10.65, *p* = 0.003), Pianp (F_(1,27)_ = 5.693, *p* = 0.0243), mGluR6 (F_(1,27)_ = 4.402, *p* = 0.0454), CaMKK2 (F_(1,28)_ = 9.188, *p* = 0.0052) and Ppp3r1 (F_(1,26)_ = 5.496, *p* = 0.027). Once again, all these differences were observed exclusively between oA*β*_1−42_-treated males and females, with no significant differences in the vehicle-treated groups.

### GSK-3*β* expression and activity alterations due to early amyloidosis treatment

Furthermore, as discussed hereafter, many of the altered protein were found to modulate, either directly or indirectly, glycogen synthase kinase-3*β* (GSK-3*β*), a protein highly involved in the regulation of LTP/LTD induction threshold. To further investigate, western blot analysis was applied to measure both GSK-3*β* expression and its phosphorylation in Ser9, which causes its inactivation.

**Fig. 5:**
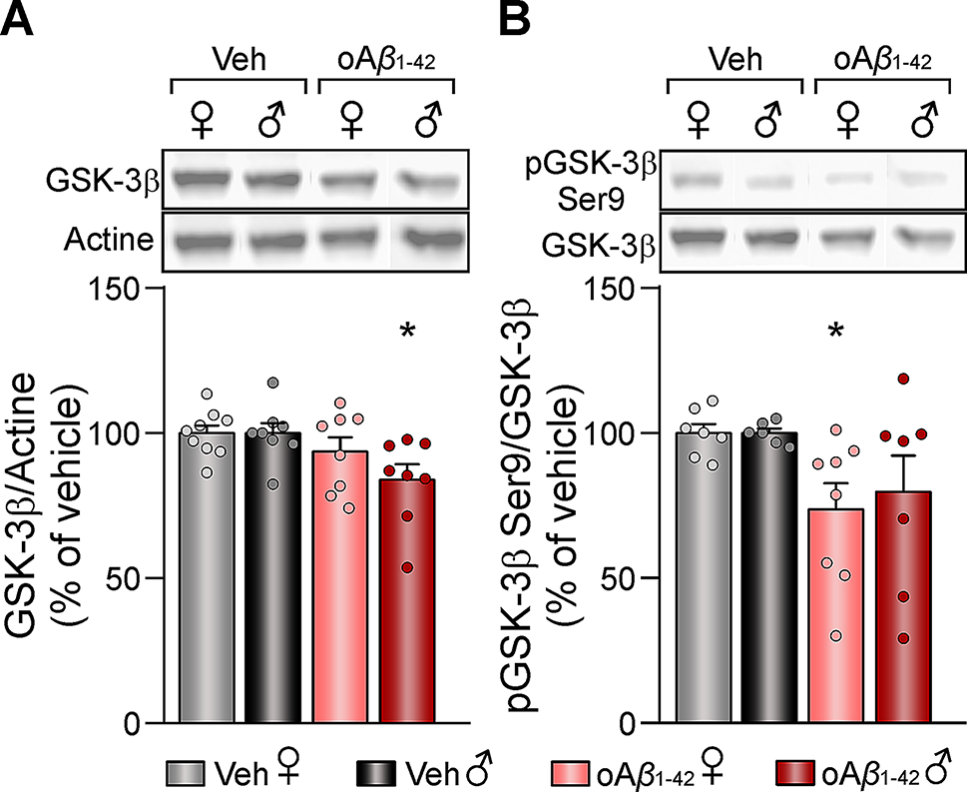
Hippocampal GSK-3*β* expression and activity levels after *icv.* oA*β*_1−42_ treatment. **(A)** Relative expression of GSK-3*β* in vehicle and oA*β*_1−42_ treated mice and representative western blots. Data are expressed as mean ± SEM of the target protein/actine as a loading control, and as percentage (%) of the control of the corresponding sex. **(B)** Relative phosphorylation of GSK-3*β* in Ser9 in vehicle and oA*β*_1−42_ treated mice and representative western blots. Data are expressed as mean ± SEM of the phosphorylated protein/total amount of protein, and as percentage (%) of the control of the corresponding sex. N vehicles: males = 6–8 and females = 7–9; N oA*β*_1−42_: males = 7–8 and females = 8. oA*β*_1−42_, Amyloid*β*_*1−42*_ oligomers; veh, vehicle; ♀, female; ♂, male. * *p* < 0.05 vs. vehicle of the corresponding sex

Regarding GSK-3*β* expression levels, two-way ANOVA revealed a significant treatment effect (F_(1,29)_ = 7.281, *p* = 0.0115) and *post-hoc* analysis showed a downregulation of this protein specifically in oA*β*_1−42_ treated males (Fig. 5A). Finally, when assessing the activity levels of GSK-3*β*, a significant treatment effect was found (F_(1,24)_ = 7.907, *p* = 0.0097), showing a lower phosphorylation of this protein in Ser9 (Fig. 5B), especially in female mice. This indicates that even though GSK-3*β* expression was reduced in oA*β*_1−42_ treated animals, the protein was overactivated due to decreased inhibitory phosphorylation.

## Discussion

The oA*β*_1−42_*icv*. mouse model of early AD has been functionally characterized by our group in both male and female individuals [14–16, 18], showing amyloid-induced impairments at different levels of functional complexity, from molecular and synaptic alterations to network activity disruptions and behavior deficits. Indeed, oA*β*_1−42_ alters hippocampal synaptic-plasticity induction threshold facilitating LTD instead of LTP, therefore inducing spatial, recognition and habituation memory deficits. Here, to further investigate early AD-like amyloid pathology at the protein level, the whole hippocampal proteome was analyzed with spatial resolution to identify possible signaling pathways/proteins underlying the deficits in LTP and spatial memory observed in this model.

MALDI imaging has emerged as an innovative technique, uniquely enabling the study of protein expression with spatial resolution directly on tissue. This study describes for the first time the main proteins modified by early amyloidosis in female and male AD mice and maps their spatial distribution in the dorsal and ventral hippocampal areas which might offer a specific proteomic signature of biomarkers of early AD-like amyloidosis and identify potential targets for early therapeutic intervention. Here, we will further discuss the results of a selection of 14 proteins found to be altered due to oligomeric amyloid treatment or sex, and that are of special interest due to their roles in synaptic plasticity and memory, as well as their modulation of GSK-3*β*, a kinase whose overactivation shifts the synaptic plasticity threshold toward LTD.

### Early amyloidosis proteome alterations

**Fig. 6:**
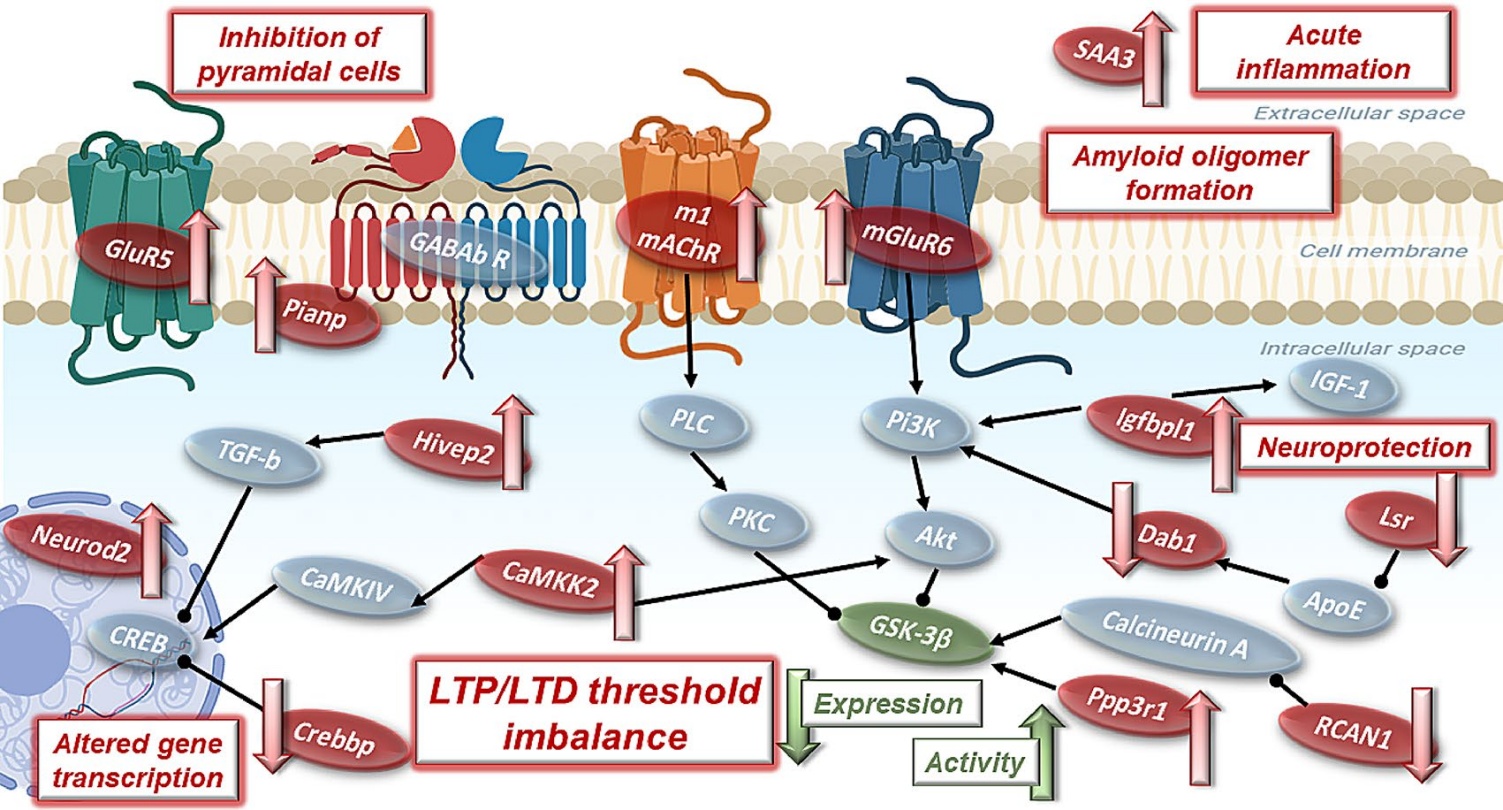
Proposed hippocampal signaling pathway altered by early amyloidosis. Proteins found to be modified by a single oA*β*_1−42_*icv.* injection are represented in red, with a red arrow indicating either up- or down-regulation, while proteins on which they exert their effect/modulate are shown in gray and connected with black lines (arrows: stimulation; dots: inhibition). Highlighted in green is GSK-3*β* due to its special relevance, as it is modulated by most of the proteins studied, and a decrease in its expression along with an overactivation have been found after oA*β*_1−42_ administration

Among those 14 proteins analyzed, our data showed a downregulation of RCAN1 and Crebbp, and an upregulation of GluR5, mGluR6, Igfbpl1 and Neurod2 in the hippocampus of both male and female mice after a single oA*β*_1−42_*icv.* injection, a well characterized model of early AD-like amyloidosis [18]. RCAN1 primarily regulates the phosphatase calcineurin, which, in turn, activates or inactivates GSK-3*β* (Fig. 6) [46], a key effector in the signaling cascade for LTP/LTD induction [47]. Accumulated evidence indicates that RCAN1 expression is chronically elevated in the brains of AD patients, contributing to the neurodegeneration characteristic of the disease [48, 49]. However, recent data indicate that both loss and overexpression of RCAN1 in the hippocampus can be detrimental, promoting memory deficits and pathophysiology similar to that observed in AD and Down syndrome patients [50, 51]. Thus, the downregulation of RCAN1 observed in oA*β*_1−42_-treated mice could partially account for the previously reported imbalance of the LTP/LTD induction threshold in this amyloidosis murine model [14, 18], without causing neurodegeneration. Moreover, Crebbp is a transcriptional regulator of CREB activity, which also modulates GSK-3*β* expression (Fig. 6) [52, 53]. In line with our results, it has been previously reported a low expression of this protein in both AD patients and murine models [52, 54, 55]. Furthermore, restoring Crebbp levels has been shown to ameliorate learning and memory deficits caused by A*β* [54], highlighting its potential as a therapeutic target in AD.

Regarding the upregulated proteins, GluR5 is a kainate receptor subtype that mediates feedforward inhibition of pyramidal cells (Fig. 6), thereby increasing the threshold for LTP induction [56–58]. Furthermore, this ionotropic receptor is also related to hippocampal *gamma* oscillations [59], which are altered in this model of early amyloidosis [16]. Another glutamatergic receptor upregulated in response to *icv.* oA*β*_1−42_ administration is mGluR6. Although this metabotropic glutamate receptor subtype has been typically associated with the retina, recent studies suggest a more widespread expression [60]. In the brain, they are critical for the maintenance of LTP by modulating the production of second messengers via G proteins (Fig. 6) [60, 61]. Thus, the upregulation these glutamatergic receptors in oA*β*_1−42_-treated mice likely contributes to the LTP deficits and subsequent robust memory impairments observed in this model [18]. On the other hand, Igfbpl1 prolongs the half-life of insulin-like growth factor-I (IGF-I) [62], which plays a neuroprotective role (Fig. 6) [63, 64]. Thus, its upregulation in the oA*β*_1−42_ group might be a compensatory mechanism to counteract A*β* neurotoxicity. Moreover, Igfbpl1 is also involved in the PI3K/AKT signaling pathway, which regulates both LTP and LTD induction [64, 65]. Finally, Neurod2 is a transcription factor involved particularly in AMPA receptor expression (Fig. 6) [66]. Elevated Neurod2 levels, as observed here after oA*β*_1−42_ treatment, have been related to defective glutamatergic synaptic formation and maturation [66]. collectively, these results points towards an alteration in GSK-3*β* ability to shift the synaptic plasticity threshold as a potential cause underlying the pathogenesis of early amyloidosis.

Notably, most of the protein expression alterations due to oA*β*_1−42_-treatment found when analyzing the whole hippocampus were located in the dorsal hippocampus, which is specifically related to spatial learning and memory [38, 40, 67], but not in the ventral hippocampus, mostly involved in emotional behaviors [38]. This might be partially explained by the fact that, after *icv*. administration, A*β* diffuses throughout the entire hippocampus but primarily reaches the dorsal hippocampal formation [14]. However, a quantitative analysis would be required to confirm this hypothesis. The only protein that showed a treatment-effect exclusively in the ventral hippocampus was M1 mAChR, which plays a pivotal role in cognitive and memory processing during sleep (Fig. 6) [68]. Moreover, this difference was observed only in male treated mice, in line with previous findings from postmortem AD brain tissue [69]. M1 mAChR is currently being targeted as a promising therapeutic strategy to improve cognitive decline in AD patients [70]. Its alteration specifically in the ventral hippocampus could indicate a role in social memory or anxiety-related processes associated with this region [38], although additional experiments would be needed to further explore this idea.

### Sexual dimorphism differences

Conversely, most of the differences between male and female mice after oA*β*_1−42_ injection were detected in the ventral hippocampus. In this area, RCAN1 and Crebbp, above discussed, were downregulated specifically in oA*β*_1−42_-treated females. Another protein less expressed in the ventral hippocampus of oA*β*_1−42_-treated females compared to males was Dab1, a key regulator of the reelin pathway (Fig. 6), which has been previously associated with trafficking and processing of APP and apoEr2 [71]. Its phosphorylation leads to the activation of the AKT/GSK-3*β* pathway and NMDA receptors [72], and both Dab1 expression and phosphorylation are diminished in the cortex and hippocampus of 3xTg-AD mice [73]. These three proteins are downregulated only in the ventral hippocampus of female mice after oA*β*_1−42_ treatment and, since this area is less affected by oA*β* in this AD model [14], these sex differences could be related to the special vulnerability of women to AD [74, 75].

Regarding oA*β*_1−42_-treated males, four proteins were upregulated in the ventral hippocampus: Pianp, mGluR6, CaMKK2 and Ppp3r1. Pianp assembles with GABA_B_ receptors, stabilizing them at the presynaptic membrane, to regulate synaptic transmission (Fig. 6) [76, 77]. This alteration may be linked to the hyperexcitability and synaptic plasticity impairments found in early amyloidosis [15, 16]. mGluR6 upregulation, as previously stated, may be related to the deficits in LTP [60]. Another key protein, CaMKK2, is crucial for late-LTP by promoting the synthesis of plasticity-related proteins and subsequent memory consolidation (Fig. 6) [78–82]. However, overactivation of this pathway has been related to synaptic loss in a transgenic AD mice model [83, 84]. Interestingly, CaMKK2 has a male-specific role in hippocampal memory formation [85], thus the overexpression of CaMKK2 in male mice after oA*β*_1−42_ injection might be contributing to LTP and memory deficits specifically in males. Finally, Ppp3r1 is the phosphatase calcineurin B, which activates GSK-3*β* (Fig. 6) [46] and a specific polymorphism in its gene have been associated with accelerated progression of AD [86–88].

In the dorsal hippocampus, only two proteins exhibited sexdifferences. CaMKK2 was the only protein overexpressed in both ventral and dorsal hippocampus, although in the former the alteration was observed in males, while in the latter, it was specific to oA*β*_1−42_-treated females. Additionally, Lsr expression was lower in oA*β*_1−42_ males compared to females. Lsr suppression has been linked to deficits similar to those reported in neurodegenerative diseases, including impairments in social and visual memory and short-term working memory(Fig. 6) [89], with a polymorphism in the gene encoding this protein being associated with a higher risk for AD [90].

Thus, it appears that in the ventral hippocampus, where less oA*β*_1−42_ seems to diffuse after *icv*. injection [14], sexual dimorphism is more evident than in the dorsal part of the hippocampus, in which most of the alterations were caused by early amyloidosis regardless the sex. It is important to note that the AD model used in this experiment reflects the earliest stages of the disease, and that the animals were mature young adults 3–6 months old. That age is considered a good representation of the preclinical stages of AD, which occur decades before disease onset [91]. During these early stages, young females benefit from neuroprotection against A*β* toxicity conferred by estrogen, which promotes non-amyloidogenic metabolism of APP and has anti-inflammatory effects [74]. However, as estrogen levels decline after menopause, this neuroprotection diminishes, potentially contributing to the increased susceptibility of post-menopausal women to AD [92]. In fact, human studies have reported a negative correlation between estrogen levels and spatial cognition [93]. Therefore, future studies should explore the spatial proteome distribution in later stages of AD using older and menopause-induced mice models.

Finally, when considering the whole hippocampus, sex-differences were observed in 2 proteins, both upregulated in males and downregulated in females after oA*β*_1−42_ treatment. The first, SAA3, is a major acute-phase protein during inflammatory responses, that is implicated in amyloid deposition and colocalizes with senile plaques in AD brains (Fig. 6) [94, 95]. Its alteration is related to the misfolding and oligomer formation characteristic of AD and other amyloidosis diseases [96, 97]. The second, Hivep2, is a protein related to intellectual disability and specifically implicated in short-term synaptic plasticity (Fig. 6) [98, 99]. It is noteworthy that different results are observed when analyzing hippocampus as a whole vs. its dorsal and ventral areas separately, stressing once again the importance of mapping of the spatial proteome distribution in AD.

### GSK-3*β*as a biomarker of synaptic plasticity alterations in early amyloidosis

Interestingly, many of the altered proteins were found to modulate GSK-3*β*, either directly or indirectly, and as revealed by the western blot assay, both its expression and activity levels were indeed altered by a single oA*β*_1−42_*icv.* injection. GSK-3β expression was reduced in male mice, while phosphorylation at the Ser9 residue, which inhibits its activity, was decreased in both females and males, leading to GSK-3β activation. This activation has been widely related to a shift in the LTP/LTD induction threshold, tilting it towards an induction of LTD [100]. Hence, the activation of GSK-3*β* in the oA*β*_1−42_-treated mice, caused at least partially by the altered proteins revealed by MALDI imaging analysis, could underlie the transformation of HFS-induced LTP into LTD previously described ex vivo [14, 18] and in vivo [15, 16] and therefore the memory deficits present in this murine model [18].

It cannot be ruled out that the observed overactivation of GSK-3*β* could act as a compensatory mechanism to counteract its reduced expression. However, such overactivation has been previously reported in AD, likely caused by the binding of oA*β* to insulin receptors, which inhibits the PI3K/Akt pathway, preventing Akt from inactivating GSK-3*β* [101]. Moreover, GSK-3*β* overactivation promotes A*β* formation through two distinct mechanisms: (1) increasing BACE1 protein levels via upregulation of NFK-β signaling, ultimately resulting in BACE1 mediated-APP processing and Aβ production; and (2) modulating γ-secretase activity by directly interacting with and regulating of PS1 activity and cellular localization [102]. Thus, GSK-3*β* has become a potential therapeutic target that deserves further investigation.

### Perspective and significance

A recent work has shown potential biomarkers related to neurodegeneration in the cortex of 5xFAD mice using proteomic MALDI imaging [37]. However, to the best of our knowledge, this is the first work that provides a mapping of the spatial distribution of the hippocampal proteome, which is of special relevance for next-generation in vivo modeling of AD [103]. The spatial mapping of the dorsal and ventral hippocampal proteome facilitated here might provide a specific signature of biomarkers of AD’s early stages. Nevertheless, a recent proteomic analysis has demonstrated a regional heterogeneity in the protein expression of the different hippocampal subfields (i.e. CA1, CA2, CA3 and dentate gyrus) in healthy humans [104], and in the context of AD, lipid expression changes seem to be specific to the CA1 subfield of the hippocampus [105]. Since each subfield is attributed a different function [106–109], the question arises whether similar results to those presented here would be observed regarding synaptic plasticity-related proteins if the hippocampal proteome of each subfield is analyzed independently. Hence, future MALDI imaging studies are needed to map the spatial proteomic distribution and changes caused by AD-like amyloidosis in the different hippocampal subfields and its implications for early diagnosis and treatment.

## Conclusions

In summary, our results key protein expression alterations related to memory formation and the underlying LTP/LTD processes in both male and female mice after a single *icv.* oA*β*_1−42_ injection [18] (Fig. 6), without evidence of neurodegeneration. These findings endorse the use of this model as a robust tool to study the early stages of AD, when neurodegeneration is not yet present. Furthermore, GSK-3*β* arises as a promising biomarker of aberrant plasticity and memory caused by early amyloidosis. Future studies should aim to further validate GSK-3β’s role and explore its potential as a therapeutic target in early AD interventions.

## Electronic supplementary material

Below is the link to the electronic supplementary material.

Supplementary Material 1

## Data Availability

The datasets generated and/or analyzed during the current study are available from the corresponding authors on reasonable request.

## Abbreviations

AD: Alzheimer’s disease
APP: Amyloid precursor protein
Aβ: Amyloid-β
oAβ_1−42_: Amyloid-β oligomers
E/I: Excitatory/inhibitory
GSK-3β: Glycogen synthase kinase-3β
Icv: Intracerebroventricularly
IGF-I: Insulin-like growth factor-I
LTD: Long-term depression
LTP: Long-term potentiation
MALDI: Matrix-assisted laser desorption/ionization
MS/MS: Tandem mass spectrometry
PBS: Phosphate-buffered saline
PPI: Protein-protein interaction
