# Supplementary material for: Mapping the hippocampal spatial proteomic signature in male and female mice of an early Alzheimer’s disease model
Source: Biol Sex Differ. 2025 May 25;16:36. doi: 10.1186/s13293-025-00697-5 (PMC12103767; doi:10.1186/s13293-025-00697-5)
Supplement: Supplementary file 1 — Supplementary Material 1 [file 13293_2025_697_MOESM1_ESM.docx]

**Supplementary file 1:**

### **Proteins validation by western blot**

To validate the expression changes identified by MALDI imaging analyses, Western blot analyses were conducted for one upregulated protein (GluR5) and one downregulated protein (RCAN1), both selected based on the availability of validated commercial antibodies.

RCAN1 was down-regulated according to MALDI imaging analysis and western blot revealed a significant treatment effect (F_(1,28)_ = 9.776, *p* = 0.0041). Although no difference between males and females was observed (F_(1,28)_ = 0.4843, *p* = 0.4922), *post hoc* analysis revealed that the effect was specifically in male oA*β*_1-42_-treated mice (Figure S1A).


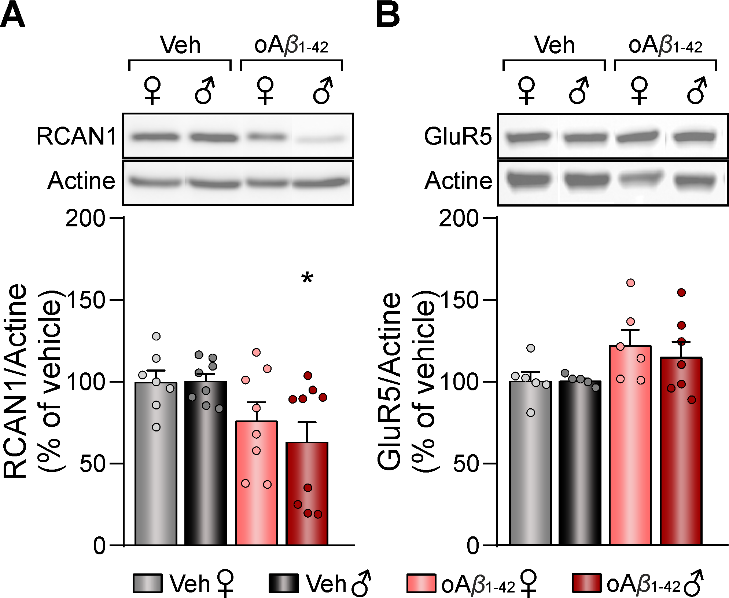
On the other hand, GluR5 was found to be up-regulated by MALDI imaging analysis as well as by western blot (treatment effect: F_(1,20)_ = 5.721, *p* = 0.0267). However, *post hoc* analysis did not indicate to which specific group the effect was due. Once again, no significant sex differences were found (F_(1,20)_ = 0.2243, *p* = 0.6409; Figure S1B).

**Figure S1.** **Validation by western blot of hippocampal proteins up- and down-regulated by oAβ_1-42_ treatment.** Relative expression of RCAN1 **(A)** or GluR5 **(B)** in vehicle- and oA*β*_1-42_-treated mice and representative western blot images. Data is expressed as mean ± SEM of the target protein normalized to actine as a loading control, and as percentage (%) of the control (vehicle) group of the corresponding sex. N vehicles: males = 5-8 and females = 6-7; N oAβ_1-42_: males = 7-9 and females = 6-8. oA*β*_1-42_, Amyloid-*β*_1-42_ oligomers; veh, vehicle. * p < 0.05 *vs.* vehicle of the corresponding sex.
